# Staff experience of a Canadian long-term care home during a COVID-19 outbreak: a qualitative study

**DOI:** 10.1186/s12912-022-00823-3

**Published:** 2022-02-21

**Authors:** Lillian Hung, Sophie C. Yang, Ellen Guo, Mariko Sakamoto, Jim Mann, Sheila Dunn, Neil Horne

**Affiliations:** 1 University of British Columbia School of Nursing, Vancouver, Canada; 2 University of British Columbia, Vancouver, Canada; 3 Community Engagement Advisory Network (Vancouver Coastal Health), Vancouver, Canada

**Keywords:** COVID-19, Outbreak, Long-term care, Qualitative research, Staff experience

## Abstract

**Background:**

COVID-19 has significant impact on long-term care (LTC) residents and staff. The purpose of this paper is to report the data gathered during a COVID-19 outbreak in a Canadian LTC home regarding staff experiences, challenges, and needs, to offer lessons learned and implications.

**Methods:**

A total of 30 staff from multiple disciplines participated in the study, including nurses, care workers, recreational staff, and a unit clerk. Focus groups (*n* = 20) and one-on-one interviews (*n* = 10) were conducted as part of a larger participatory action research (PAR) study in a Canadian LTC home. All data collection was conducted virtually via Zoom, and thematic analysis was performed to identify themes.

**Results:**

Four main themes were identified: We are Proud, We Felt Anxious, We Grew Closer to Residents and Staff Members, and The Vaccines Help.

**Conclusions:**

This research details the resilience that characterizes staff in LTC, while highlighting the emotional toll of the pandemic, particularly during an outbreak. LTC staff in this study found innovative ways to connect and support residents and this resulted in stronger connections and relationships. Leadership and organizational support are pivotal for supporting team resilience to manage crisis and adapt positively in times of COVID-19 pandemic, especially during the period of outbreak.

## Introduction

COVID-19 has taken an enormous toll on long-term care (LTC) residents worldwide. As of mid-2020, over 80 percent of COVID related deaths were LTC residents [1]. Canada has a total of 2,076 long-term care homes; British Columbia (BC) has a total of 308 long-term care homes [2]. The majority of residents (80%) are living with cognitive impairments, who also have complex and multiple co-morbidities [3]. Although LTC homes serve high-risk and vulnerable populations, they do not share the same resources as other health care settings [4]. While there is evidence that many LTC facilities have reported experiencing personal protection equipment (PPE) and staff shortages [5], LTC staff have also experienced growing anxiety and stress throughout the pandemic, particularly during virus outbreaks [6].The World Health Organization (WHO) (2021) underscored that careful attention should be paid to protect people living and working in LTC homes [7]. This includes acknowledging the ongoing value and contribution of older people in society [8], including those who live in LTC [9]. LTC homes are ubiquitous in Canada and provide important locales of care for people who can no longer remain in their own homes. While there has been considerable public concern regarding the impact of the pandemic on residents living in LTC, attention has not been focused on lessons learned from LTC staff themselves, particularly during COVID-19 outbreaks. Knowledge generated from the voices of staff is necessary to inform change and practice development for safety and quality of care.

### Background/ context

Staff in LTC settings need knowledge, skills, and a person-centred care culture to ensure positive experiences for residents. Helping residents to eat well is essential for meeting physical, emotional, and social needs [10]. In 2019, we began a study (Happy2Eat) to engage residents, families, staff, and leaders to identify priority needs and strategize actions to promote person-centred care at mealtimes. The study took place in a 250-bed publicly funded Canadian LTC home on British Columbia’s west coast. The resident population is multicultural and has a complex level of needs, requiring 24-h nursing care for chronic diseases/disabilities. Over 85% of residents have cognitive impairment or dementias.

In the first six months of the Happy2Eat study, we co-developed practical strategies in a LTC home to enhance the dining experience of residents with dementia through regular weekly staff huddles. Frontline staff (nurses, care workers, dietary staff, rehabilitation, and recreational staff) gathered for team reflections and explored creative ways to promote person-centred care. For example, staff reorganized meal routines and spent more time to provide mealtime care according to individualized needs. In early 2020, the COVID-19 pandemic put the project on hold for 9 months. In the fall when the research resumed, a COVID-19 outbreak was declared at the study site. The residents and staff in the LTC care home experienced high anxiety and many challenges. Family visits were banned. Following the outbreak, restrictive infection control policies and regulations significantly impacted how the staff practiced their care in the home. Frontline staff stepped into the family’s role to support residents’ emotional and nutrient needs by fostering close relationships with residents and decreasing residents’ sense of loneliness and isolation during the facility lockdown [11]. This paper aims to report specifically on the staff experience of caring for residents during a COVID-19 outbreak in 2020. Here we offer lessons learned and practical implications to inform practice development.

## Methods

### Study design

This was qualitative descriptive design [12]. Person-centred care theory guided the study, recognizing care practices that promote personhood, rather than care that is merely task-focused [13]. We worked with patient partners and clinicians closely in the research because as practice in LTC is highly complex, a collaborative approach is required to make and sustain change [14, 15]. The larger study (Happy2Eat) is a Collaborative Action Research; we engaged frontline nurses and staff in all disciplines to identify both the challenges and practical strategies to improve residents' dining experiences [15]. The Happy2Eat study was carried out through three phases: (1) Looking (observation and focus groups), (2) Thinking and Acting (strategizing and taking action for change), (3) Evaluation (interviews and focus groups to assess impacts). We have completed the first phase and actively worked with staff during phase 2. Staff had regular team reflection about challenges and creative solutions. Due to the significant impact of the COVID-19 outbreak, staff spoke extensively about their experience of caring for residents during a COVID-19 outbreak. Thus, this article reports timely data gathered at phase 2 (i.e., staff experience discussed in focus groups and individual interviews).

### Recruitment of participants

A convenient sampling method was used for recruitment. The sample included 30 staff (including nurses, care aides, and dietary staff). Separate posters were posted to invite staff participation for focus groups and individual interviews. The local nurse leaders also sent out group emails to all staff to help recruit participants. The inclusion criteria were staff working full time or part-time at the study site, being willing to participate in the study, and working in the frontline to provide direct care for residents, including those with COVID-19. ﻿There was no specific exclusion criterion. ﻿The characteristics of the participants are reported in Table 1.

**Table 1:** Characteristics of the participants

Participants	Sex	Discipline	Years of work experience
P1	Female	Nurse	3
P2	Male	Nurse	5
P3	Female	Nurse	6
P4	Female	Unit clerk	2
P5	Female	Nurse	10
P6	Female	Care worker	10
P7	Male	Care worker	11
P8	Female	Care worker	12
P9	Female	Care worker	10
P10	Male	Care worker	11
P11	Female	Nurse	8
P12	Female	Care worker	6
P13	Female	Care worker	7
P14	Female	Care worker	9
P15	Male	Care worker	11
P16	Female	Nurse	2
P17	Female	Nurse	1
P18	Female	Recreation staff	1
P19	Female	Recreation staff	1
P20	Female	Nurse	2
P21	Female	Care worker	8
P22	Male	Care worker	9
P23	Female	Care worker	7
P24	Female	Care worker	10
P25	Female	Care worker	15
P26	Female	Care worker	22
P27	Female	Care worker	26
P28	Female	Care worker	12
P29	Male	Care worker	6
P30	Female	Nurse	1

### Data generation

We conducted two focus groups (*n* = 20) by Zoom meeting and individual interviews (*n* = 10) by phone to investigate critical challenges, experiences, and support needed for frontline staff in a long-term care home. A total of 30 staff in multiple disciplines participated in the study. They included Registered Nurses, Licenced Practical Nurses, care staff, recreational staff, and unit clerks. The Primary Investigator moderated two focus groups. The focus group sample composed of a total of six nurses, ten health care aides, one unit clerk, and one recreation worker. ﻿Participants were informed of the interview questions a week before the focus group interview to provide them an opportunity to consider and reflect on the questions in advance. In the focus groups, we asked: What was your experience about caring for residents, helping them to eat? What are the challenges? What resources do you need for best support? The focus groups were conducted virtually through zoom meetings in December 2020, each lasted for approximately 60 min. The semi-structured telephone interviews were conducted by phone in January 2021, each lasted for approximately 30–40 min. We asked: What is it like to care for residents during the outbreak? Tell me about a challenging time when you helped a resident with COVID-19 to eat? Both focus group and interview data were recorded and transcribed verbatim.

### Data analysis

We performed thematic analysis to identify themes [16]. The analysis was led by LH and SY and the procedures were completed in three steps. First, all authors read and re-read the data in the transcriptions independently to gain a familiarity of the content. Second, LH and SY manually searched for the codes and patterns across the data and identified initial themes. Both inductive and deductive approaches were used. While the data set was preliminary coded inductively, concepts based on the literature in person-centred care theory were also used for deductively coding. For example, “Proud” was an inductive code used to capture the positive emotion in the team. “Relationship” was a sensitized concept, a deductive code, informed by the literature in person-centred care theory. This process involved going back and forth between the data and the literature. The third step of analysis involved team analysis with all authors (including patient and family partners, as well as clinician researchers) to discuss the themes together in research meetings and gain analytic consensus. Our team analysis focused on reviewing, validating and refining the final themes.

### Trustworthiness

We used a reflexive approach to enhance trustworthiness of the study. ﻿ Our team reflection allowed us to discuss and compare our own assumptions and paid attention to how assumptions might influence our thinking and actions. Research rigour was ensured by careful guidance by the academic supervisor and regular debriefings with the whole team, including patient and family partners. ﻿ We took notes to capture analytical thoughts and iterative analyses. ﻿ To establish dependability, we offer a rich description of study context and methods. For credibility, including patient and family partners in the research team helps to demonstrate both a recognition that knowledge exists beyond a single perspective and the alignment with principles of participatory action research [17]. Our team reflexive meetings helped bring a shared awareness of the complexity of clinical situations and staff experiences.

### Ethics

The study was approved by the Research Ethics Board at the University of British Columbia and the local health authority. ﻿All staff participants gave written informed consent to participate in the focus group discussion and individual interviews. A small token of appreciation ($15 coffee card) was provided for each participant.

## Results

Our analysis identified four themes that highlight experiences reported by frontline staff caring for residents in long-term care during a COVID-19 outbreak. Hearing the voices of staff provided an opportunity to document their perspectives. In focus groups and interviews, many staff thanked us for listening to their voices – the interpretations of their own stories and experiences in the care home. They had much to say about the challenges they faced, their personal experiences – both the positive and the very painful, and the useful supports that helped.

The pandemic impacted the staff and residents in significant ways with intense and complex emotions. Table 2 shows the themes.

**Table 2:** Common Themes of LTC Staff Experience During a COVID-19 Outbreak

Theme	Subthemes
**“We are proud”**	Resilience, growing stronger
	Passion for their role
**“We felt anxious”**	Safety & safety protocols
	Resident mental health
**“We grew closer together”**	Residents
	Staff
**“The vaccines help”**	Protection against outbreak & increased confidence for safety
	Return to old routines

### “We are proud”

#### Resilience, growing stronger

This theme describes how the staff expressed pride in their resilience amidst tough times of high stress and challenging experiences. They felt proud for stepping up to the challenge, taking ownership of their duties, and persevering. Growing stronger and feeling prepared to tackle future hardships were common themes. For example, one nurse commented:

Well, we lost a lot of lives … but we always think about the brighter side. At least I do. This is something we can’t control. We just do what we have to and whatever happens, it’s a good experience for us. I feel sorry for those who lost their lives and their families, but life must go on. We’re still standing up. And I think, standing stronger now!

Interestingly, several participants used the language “a good experience” to refer to the outbreak. The resilience in the frontline staff was evident and helped the team overcome hardship and adversity. Staff reframed the ordeal into a learning experience demonstrating their positive attitude to cope with stressors. Their shared resilience served as a source of empowerment to keep individual staff member passionate about their professional role for residents in the care home.

#### Passion for their role

Participants displayed clear passion for their work, reflecting on the sense of purpose they derived from serving the residents. They took pride in their efforts in caring for the residents with high vulnerability. For example, a care worker recounted that she felt so gratified when witnessing the recovery of residents who were infected by COVID-19.

Knowing them all, I was quite touched when I saw [the residents] starting to go out of their rooms and those patients who were positive… We thought they really couldn’t make it. Now, they’re out in the dining room being their normal selves again. It’s quite rewarding! ... It’s a really nice feeling thinking about how hard we worked for them, and [how] it actually worked …

One nurse explained their perception about their professional identity and how that mentality boosted their team spirit and capacity to cope with adversity.

We signed up for this job. This is actual nursing! Sometimes we get burnt out, right? So busy, and here’s the supervisor asking us to do this and that. It is difficult and risky work. But we signed up for this. It’s like the army. Now you go to war. It’s the real deal. We got to do what we need to do.”

One care worker added, “I was the one who discovered [this resident] with a very warm temperature. I did proper PPE, always made sure she was eating and drinking. Then she got better and recovered.” In all of the interviews and focus groups, staff displayed a strong desire to tell their stories, and described feeling valued and recognized for being listened to. They spoke with conviction about their roles and their desire to celebrate their work.

### “We felt anxious”

#### Safety & safety protocols

Psychological stress from the possibility of contracting or spreading COVID-19 was common amongst participants. Additionally, routine tasks such as mealtimes came with the added responsibilities of safety procedures such as changing Personal Protective Equipment (PPE) every time they entered and exited a resident’s room. Staff members spent considerable energy to meet these demands. A care worker said, “It’s like we had a fear inside because we have families too, and other residents. Another nurse described how she met the challenges with compassion and positivity when helping sick residents:

I tried to make sure I did everything right… But sometimes they cough or spit a little bit, I have to be close to help them to eat and drink; I can’t just run away. I asked them to raise their hand when they need to cough, so I move back or to the side a little. Sometimes, it’s bit scary to some of us. But we have lots of PPE available and we just make sure to take the time to follow the procedures.

We also heard staff participants telling us about being selfless, brave and courageous. Some admitted that they were more worried about the vulnerability of residents and less worried about personal safety. This finding is surprising but revealing. It exposes this instinctual tendency of many frontline staff: care for residents first, think about personal consequences later. For example, a care worker in the focus group stated, “We are not really scared of getting the virus from the residents. We are more worried about me giving it to [residents]”. A nurse commented, “I feel confident with the PPEs but I also have an attitude that I don’t live my life worrying about how I might get ill”. While it was evident that the availability of PPE and resources raised confidence in their ability to work safely, staff indicated that ongoing education and support are required to support staff to work safely in the LTC environment. Many participants reported that they felt supported by timely education needed for safety and quality of care. A care worker described it well:

Everyone was anxious and scared ... but the infection control team taught us about what COVID is, how it spreads, and how we can protect ourselves. That gave us confidence going through the difficult situation.

#### Resident mental health

Participants chronicled the heartache of watching the residents’ mental health decline. During the outbreak, residents were required to stay in their rooms and forgo activities such as having meals and coffee in dining rooms or going for walks around the facility. Staff saw the effects that social isolation and disruption of routines had on the residents – many of them experienced distress, confusion, and loneliness, in addition to weight loss and frailty. Comforting residents of varying cognitive abilities was challenging. A care worker explained the traumatic impact:

I’ve had residents tell me they feel like a prisoner, like “What did I do wrong?” You try to explain to them, “No, you didn’t do anything wrong.” I remember one resident in the beginning, long before the outbreak happened. He was sitting by the window one day and goes, “Is this virus still out there?” and I go, “yeah, unfortunately it’s still out there,” and he goes, “But I can’t see anything!” That just broke my heart.

“Broken heart” was a phrase we heard repeatedly in the staff interviews. Staff described the pain of seeing residents deteriorating with prolonged confinement and limited social stimulation. One recreational staff member expressed sadness and guilt, “I would leave there some days with my heart broken because I’m thinking, they’re stuck in a 5 by 5-foot room. I could leave there but they didn’t have freedom”.

Staff described how residents with dementia did not understand the pandemic situation. Many residents felt they were abandoned by family because of the ban on visitors. Witnessing the distress in residents was hard for the staff. For example, a nurse recalled that a resident used to going out for meals with her son, said, “My son left me here! He is not coming to see me.” The nurse explained about the pandemic restrictions, but then the resident would forget and remained in distress.

There were many similar sad stories in the interviews. A care worker reported,

There was one resident who passed away because of COVID. Other residents got really depressed. It was hard to see them saying that they were waiting to die and lose hope, “When am I going? What’s happening to me?” They need more encouragement, so you talk to them. You have to make them feel better.

Empathy is evident in the staff’s voices. While empathy is a requisite for comforting residents, it often comes at a cost to their own wellbeing. Despite the stressors related to safety and mental health, they consistently showed compassion to residents.

### “We grew closer to residents and staff members”

#### Residents

Staff members felt that relationships with residents grew stronger. Many participants attributed this to the extra staffing provided by the organization during the outbreak, which enabled them to provide physical and psychosocial care with residents. A nurse explained her experience and illustrated the need for social engagement,

When everybody was taken care of, we had more time to interact with the residents.

I noticed [that] when I have time to talk to them, they will tell me the story of their life and it’s really interesting. They really want to talk.

Mealtimes were an important time of socialization. Prior to the pandemic, loved ones often visited to help feed the residents, but this responsibility fell on the staff during the outbreak. They helped fill social gaps from absence of family, volunteers, and other resident interactions. These interactions provided opportunities to get to know the residents in ways that they previously did not have before. A recreation staff said, “some of the residents’ families always come to feed—but [now] we had the chance to help them to eat. Now we know them more and feel more attached to them.” One care worker gave an example of his work during the outbreak period.

One of my residents, he likes to talk. I go in in the morning, we have breakfast, and he usually asks me “How does it look outside?” I open the curtain and tell him it’s a nice day, or it’s rainy. He says, “I can’t go out?” and I say, “Not for now.” so I just hold his hand. He has a cellphone: I helped him call his wife.

Working in a multicultural facility, staff members found that using residents’ own languages and culture to connect was an effective way to boost their spirits and put them at ease. One nurse participant talked about their attempts to learn the language of the residents; another nurse talked about connecting staff and residents with similar backgrounds. “I don’t speak the language, but I try to learn a few words of Cantonese. That puts joy on the residents’ face. It is nice to see the joy in residents when I’d put on a Chinese drama for them”.

#### Staff members

Similarly, staff members grew closer to one another. Keeping the same people on each unit during the outbreak helped build community. They mentioned having daily team meetings where they shared their experiences and the strategies that worked for them that day. There was a sense of solidarity to provide the best, safest care possible while looking out for one another. A care worker explained, “if you worked in one unit, you couldn’t work in another unit because of the infection prevention policies. We worked more shifts because of the staffing need. That made the team close!” A nurse described how they worked together to provide care, “if a care worker was in a room to give care with full PPE, we would say “Okay, no worries, just call me and I’ll bring you everything.” A care worker underscored, “before, care aides and nurses had many conflicts. But right now, we’re like 'Don’t think about that, we have to help each other to get through this' ”. Staff members felt that the strengthened connections with residents and other staff members was a main positive outcome. They described the heightened level of teamwork as crucial to their success.

### “The vaccines help”

#### Protection against outbreak & increased confidence for safety

This large public facility (owned and operated by the health authority) was amongst the first in their jurisdiction to receive vaccinations. At the onset of the outbreak, staff and residents were prioritized to be immunized. Many staff perceived that the vaccine helped cushion the effects of the outbreak and kept the outbreak relatively well-controlled, compared to other smaller LTC homes with less support and resources. A nurse reported, “I think the vaccine helped the residents because up to the time we discovered the last case, we maintained that number and didn’t have more after that”. Consistently, staff reported that having most residents and staff members immunized helped them feel safe to work. For example, a care worker said, “we all had a first dose, the residents and us. It gives you confidence. You have peace of mind that you’re protected”.

With the protection of the vaccines, the facility loosened the restrictions once the outbreak ended, such as permitting residents to leave their rooms and allowing for some family visits. This came as a relief to many residents after spending over a month confined to their rooms and was a positive and important step by staff towards a return to normalcy.

## Discussion

The findings of this research provide important insight into what was occurring in LTC homes during a COVID-19 outbreak. To date there has not been much investigation into the experiences of staff during this critical and stressful time, when a care home goes into lockdown. While the pandemic has laid bare the many long-standing issues that exist in LTC, it is also vital to underscore the important work and effort of LTC staff during this time, as well as the positive lessons learned. The voices of frontline staff provided a detailed description of their emotional experiences and the positive stories about caring for residents with dedication and compassion. What is unique in the results is the resilience frontline staff displayed for resident care despite overwhelming emotion and risky challenges. Resilience is a concept often discussed in workplace literature, particularly in the context of health care workers’ capacity to cope with the negative effects of stress while striving to maintain mental wellbeing [18]. It is important to point out that resilience is a dynamic concept and can be increased and lowered in the team. In the study, participants described how structural and interpersonal support boosted their capacity to adapt and persevere from unprecedented challenges. Staff burnout can have a heavy toll on care work. More research is needed to further investigate how team resilience can be built and nourished by structural support and policies for a healthy and safe workplace.

The impact of building relationships is another important piece that has come out of this work. Staff noticed that they were able to build stronger relationships with residents, in part because they had to connect with them more frequently without family there, and also because staffing levels were augmented at the study site during the outbreak. This shows the need for increased and adequate staffing in LTC moving forward, and for society to critically reflect on how best to equitably support the LTC sector once the pandemic is over. Pre-pandemic, family members played an important role in supporting residents with their daily needs [19]. While it will be crucial to embrace family members back into existing care teams, it will be equally essential for there to be recognition of the need for ongoing staffing that is adequate, even once the COVID-19 crisis has resolved. This is particularly important as we know that Canadian seniors who have close relationships with LTC staff members have a relatively lower risk of mortality [20]. It is important to note that staff in the study have learned person-centred care principles (e.g., putting the person first, paying attending to psychosocial needs and relational practice, avoiding task-focused) and practiced regular team huddles in the Happy2Eat project. It is possible that the knowledge and skills of person-centred care have empowered staff to have a greater capacity to undertake their work. It is encouraging to see that participants spoke of the effort they took during the outbreak to engage with residents in person-centered ways. In addition, building and strengthening working relationships amongst staff members was critical in getting through the outbreak. Notably, it seemed that hierarchical structures, which can be problematic in health care [21], were flattened as nurses, care aides and other staff worked together to support one another and to ensure that residents were cared for. Future studies should investigate how person-centred care training may help staff build team resilience for positive mental health and mitigate traumatic impacts during challenging and difficult times.

The World Health Organization (2020) has warned about the potential negative impact that the pandemic has had on direct-care staff’s mental wellbeing [22]. Globally, many health care workers have spoken about the trauma that this pandemic has incurred in their lives, such as the experience of losing patients, and the stress of being at the frontlines [23]. Health care workers can experience moral injury, which can lead to burnout and other mental health concerns [24–27]. Findings from this study revealed the psychological stress and anxiety that participants felt at the possibility of contracting or spreading COVID-19 because they provide personal care, with regular prolonged close proximity with residents [28]. This research has practice and policy implications for better ongoing mental health and psychosocial support in LTC. National LTC standards and guidelines should emphasize greater protection for staff to ensure a safe and healthy work environment.

When it comes to infection control, evidence shows that many LTC facilities are still experiencing PPE shortages [5, 29–31]. Ensuring ongoing and adequate PPE, training on how to use it properly, and organizational support for LTC staff will be critical for alleviating anxiety, contributing to safety. The importance of the vaccines as a public health measure is noteworthy, especially as early vaccination efforts focused on the LTC sector and worked to protect residents and staff. Many of the staff who participated in this study were optimistic about where things were headed, especially as vaccination efforts were prioritized in LTC. They also felt valued and recognized when they received timely and needed resources for staffing, education, and material support.

### Strengths and limitations

This study was conducted against the background of the outbreak of COVID-19 so our timely analysis provided useful insights to inform practice and support resident care. Similar to other research studies conducted during the COVID-19 pandemic, ﻿in person access to the LTC home and staff was impossible [32]. Virtual focus groups and phone interviews limited researchers’ capacity to read participants’ emotional expression. In the Zoom focus groups, staff wore facial masks and face shields. While zoom and phone interviews avoided the risk of infection, the remote connection did not allow the interviewers to read body gestures which can convey important information. Subtle body language cues are much easier to pick up in in-person observation and interactions, which could have provided in-depth understanding of contextual details and circumstances.

It is important to note that the staffing at the study home was stable, as the site is well resourced and supported being a large owner-operated facility. During the pandemic, British Columbia was the first province in Canada ordered the single-site policy that prohibited staff to work across LTC homes. This may have contributed to the findings in this study, particularly in regard to the resilience found amongst staff members and the strength of the relationships formed, as they had a consistent team working together throughout the outbreak.

In this study, one third of the participants had three or less years of experience. This may have increased the sense of relying on and supporting one another, given the urgency of the outbreak situation and the need to come together to adequately care for residents. Future research should examine staffing levels and years of experience in outbreak situations to better understand whether these variables impact care, and resident and staff experiences.

## Conclusion

This study has revealed the important work of caring during an outbreak, bringing much needed attention to the valuable work in the LTC sector. Positive relationships and good care are possible during challenging times if staff are well supported. LTC staff in the study felt empowered by what they achieved during the pandemic. Their stories demonstrated that the work that they do counts and that the efforts they have undertaken have made a difference. Good care relies on a healthy workforce that is resilient to adapt. Moving forward, it is paramount to have better policy and structural support to nourish resilience, promoting mental health and wellbeing of LTC staff. Health care leaders, policy makers, and all members of society will need to act on lessons learned during the COVID-19 pandemic to ensure that the LTC setting is a safe and supported environment for health care workers to work in, as well as for the residents who live here.

## Data Availability

Dr. Lillian Hung may be contacted if someone would like to request the data.

## Abbreviations

LTC: Long-term care
PAR: Participatory action research
CAR: Collaborative action research
PPE: Personal protective equipment
